# Microbial co-occurrence networks of gut microbiota reveal community conservation and diet-associated shifts in cichlid fishes

**DOI:** 10.1186/s42523-020-00054-4

**Published:** 2020-09-29

**Authors:** Joan Lluís Riera, Laura Baldo

**Affiliations:** 1 Department of Evolutionary Biology, Ecology and Environmental Sciences, University of Barcelona, Barcelona, Spain; 2 Institute for Research on Biodiversity (IRBio), University of Barcelona, Barcelona, Spain

**Keywords:** Bacterial association, Microbial communities, Lake assemblages

## Abstract

**Background:**

The extent to which deterministic rather than stochastic processes guide gut bacteria co-existence and ultimately their assembling into a community remains largely unknown. Co-occurrence networks of bacterial associations offer a powerful approach to begin exploring gut microbial community structure, maintenance and dynamics, beyond compositional aspects alone. Here we used an iconic model system, the cichlid fishes, with their multiple lake assemblages and extraordinary ecological diversity, to investigate a) patterns of microbial associations that were robust to major phylogeographical variables, and b) changes in microbial network structure along dietary shifts. We tackled these objectives using the large gut microbiota sequencing dataset available (nine lakes from Africa and America), building geographical and diet-specific networks and performing comparative network analyses.

**Results:**

Major findings indicated that lake and continental microbial networks were highly resembling in global topology and node taxonomic composition, despite the heterogeneity of the samples. A small fraction of the observed co-occurrences among operational taxonomic units (OTUs) was conserved across all lake assemblages. These were all positive associations and involved OTUs within the genera *Cetobacterium* and *Turicibacter* and several OTUs belonging to the families of Peptostreptococcaceae and Clostridiaceae (order Clostridiales). Mapping of diet contribution on the African Lake Tanganyika network (therefore excluding the geographic variable) revealed a clear community change from carnivores (C) to omnivores (O) to herbivores (H). Node abundances and effect size for pairwise comparisons between diets supported a strong contrasting pattern between C and H. Moreover, diet-associated nodes in H formed complex modules of positive interactions among taxonomically diverse bacteria (mostly Verrucomicrobia and Proteobacteria).

**Conclusions:**

Conservation of microbial network topologies and specific bacterial associations across distinct lake assemblages point to a major host-associated effect and potential deterministic processes shaping the cichlid gut microbiota. While the origin and biological relevance of these common associations remain unclear, their persistence suggests an important functional role in the cichlid gut. Among the very diverse cichlids of L. Tanganyika, diet nonetheless represents a major driver of microbial community changes. By intersecting results from predictive network inferences and experimental trials, future studies will be directed to explore the strength of these associations, predict the outcome of community alterations driven by diet and ultimately help understanding the role of gut microbiota in cichlid trophic diversification.

## Background

The gut microbiota composition is determined by both the environmental microbial exposure and the host intestinal environment, which imposes strong constraints in the colonization and mediates community assembling through specific niche offering [1–3]. The combined influence of the host and the environmental mediated factors (e.g. dietary inputs) can therefore result in specific and to some extent predictable gut communities [4–6]. Still, the extent to which deterministic rather than stochastic processes guide microbes co-existence and ultimately their assembling into a community remains a matter of debate [3, 7].

Co-occurrence networks are a powerful tool to start exploring the forces that affect gut microbial community structure and its dynamics [8]. This increasingly used analytical tool relies on microbial abundance data obtained from extensive sequencing data (typically a matrix of OTUs) to infer microbe interactions as a function of their covariation patterns across a wide number of samples [9]. The assumption is that a non-random pattern is shaped by ecological processes driving coexistence (e.g. cross-feeding or partial niche overlap) or exclusion (e.g. competition or predation) [9, 10].

Comparative analyses of microbial networks built from distinct datasets that vary at one or multiple traits (either host or environmental) are particularly powerful to explore microbial community dynamics [11]. This type of approach can retrieve network commonalities across systems, e.g. microbial associations that are persistent along major host/environmental-associated variables (i.e. taxonomical, spatial, temporal and ecological), as well as unveil system and trait-specific co-occurrence patterns (e.g. with the host health status, ethnicity and dietary habits) [11–15]. While not being conclusive in terms of inferences, these methods allow a first exploration beyond microbiota compositional aspects and set the ground for empirical testing of novel hypotheses [8]. Recent studies have successfully applied this comparative approach to the exploration of microbial associations at distinct scales of biological organization, i.e. across host populations [12], species [14] and within and across distinct biomes [10, 11, 13], with a major focus on humans. Little is known about how much of the observed gut microbial diversity engage into robust interactions in nature, and whether these interaction patterns are maintained or modified during the natural process of host diversification.

Cichlid fishes provide an attractive system to investigate gut microbe-microbe association patterns and community changes during host divergence. They represent an iconic fish family (Cichlidae), widely distributed across lakes and rivers in the subtropical/tropical regions and have served as a primary model to study speciation and rapid phenotypic diversification [16, 17]. The extraordinary range of ecological niches they occupy, even within highly reduced water pools (e.g. small crater lakes), are primarily driven by resource partitioning and niche displacement, following competition for local resources [18–21]. In the African Great Lakes (Tanganyika, Malawi and Victoria), repeated explosive adaptive radiations have led to the greatest levels of cichlid specialization in terms of morphology, behavior and dietary preferences [17, 22]. Central American lakes, on the other hand, host more recent and less ecologically diverse fish assemblages (all species belong to the same genus *Amphilopus* [21]. While very diverse and widely distributed, cichlids are nonetheless a relatively young family (most species have evolved within the past 0.5 My), with distinctive and unifying phenotypic traits [17], shaped by a common genomic structure [23].

Recent analyses have shown a strong correlation between compositional aspects of the cichlid gut microbiota and dietary habits, suggesting that microbes could play a role in optimizing fish digestion and therefore participating in cichlid trophic adaptation [4, 24]. These studies also revealed the existence of a small microbial component conserved across all cichlids (core). It remains unclear, however, whether these diet-associated and conserved compositional traits of the cichlid gut microbiota are driven by specific microbe-microbe interactions and how host ecology and geography influence these associations.

Here we provide the first exploration of the cichlid gut microbial associations through co-occurrence network analyses. We take advantage of a recently published large scale gut microbiota dataset [24], including samples from two African and seven American lakes encompassing major dietary niches, to address the following major objectives: a) provide the first description of the cichlid gut community network; b) identify network features and pairwise occurrences robust to major host ecological and geographical variables (i.e. the core network); c) detect diet-specific association patterns that can help us understanding microbial community changes during cichlid dietary adaptation.

To reach these goals, we partitioned the dataset according to major geographic and dietary groups and built individual co-occurrence networks using a consensus of correlation and dissimilarity metrics. We then contrasted the networks generated to retrieve commonalities/differences in microbial associations across fish assemblages. Finally, we explored the diet contribution to the co-occurrence patterns observed in Lake Tanganyika by mapping major dietary groups on the network layout.

## Results

### Comparative analyses of lake and continental networks

**Fig. 1:**
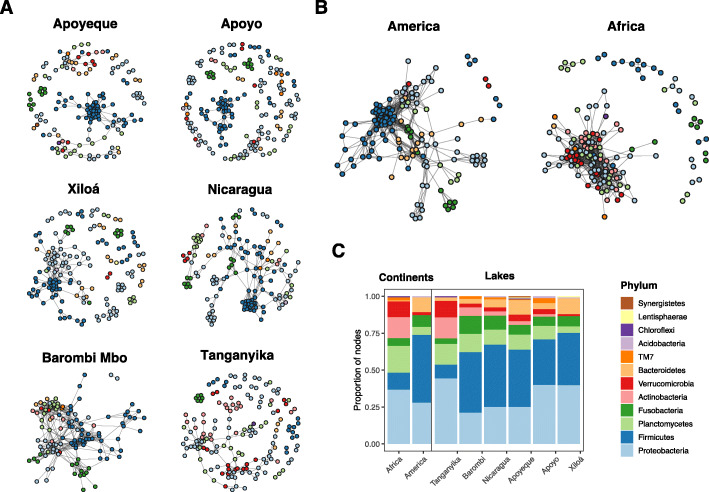
Co-occurrence network graphs of the cichlid gut microbiota and their node taxonomic composition. **a-b** Networks for individual lake assemblages (**a**) and continental datasets (**b**), where nodes are OTUs color-colored to the phylum level. **c** Proportion of nodes (OTUs) per phylum found in each geographical dataset (for the overall microbial taxonomic composition, see [24], while for proportion of OTUs per phylum in the input dataset see Additional file 4: Figure S3). All American networks largely resembled in topology and node taxonomic content. Note the dense cluster of Firmicutes nodes present in all lake networks (blue circles, in L. Tanganyika the cluster is highly reduced), encompassing the two families Clostridiaceae and Peptostreptococcaceae (for family level, see Additional file 2: Figure S1). Network interactions were inferred based on concordance among four co-occurrence measures in the ensemble package CoNet [25] and visualized with *igraph* [26]

We used the CoNet ensemble approach based on four correlation measures (Spearman, Pearson, Bray-Curtis and Kullback-Leibler) to infer robust bacterial association patterns in the cichlid gut. To this goal, we built co-occurrence microbial networks for the two continental datasets, Africa and America, and for six lake datasets, including the African Lake Tanganyika and crater lake Barombi Mbo, and the American lake Nicaragua and crater lakes Apoyo, Apoyeque and Xiloá (Fig. 1a-b).

Despite the heterogeneity of the fish data in terms of species phylogeny and ecology (42 species and five major diets, for details see Additional file 1: Table S1, and [24]), both individual lake and continental networks were largely comparable in size (number of nodes varying between 122 and 175) and global topological features (Table 1). OTUs engaging into significant associations (i.e. nodes, *p* < 0.05, Benjamini-Hochberg (BH) correction) represented between 21 and 43% of the total OTUs per dataset present in the original input matrix (Table 1; for the original matrix see Additional file 1: Table S2). The large majority of the associations (edges) were positive (copresence) for all networks. These were all relatively poorly dense (0.02–0.10, graph density) and showed comparable clustering coefficients (0.5–0.7). Modularity, as calculated by Louvain, ranged between 0.6 and 0.8 (Table 1).

**Table 1:** Global network topologies

	Dataset	N. of samples	N. of OTUs^a^	N. of nodes	N. of edges (+/-)	Mean Node Degree	Clustering coefficient	Path length	Density	N. of Modules	Modularity
Continent	America	161	716	150	1113 (1083/30)	14.84	0.69	3.34	0.10	17	0.44
	Africa	116	637	149	881 (856/25)	11.83	0.50	2.44	0.08	10	0.43
Lake	Nicaragua	24	504	147	483 (448/35)	6.57	0.71	6.03	0.05	12	0.70
	Apoyo	24	510	175	324 (321/3)	3.70	0.61	2.68	0.02	32	0.82
	Apoyeque	25	356	155	405 (370/35)	5.23	0.60	5.05	0.03	18	0.57
	Xiloá	43	494	158	364 (352/12)	4.61	0.58	3.65	0.03	29	0.70
	Tanganyika	73	597	162	276 (276/0)	3.41	0.49	5.77	0.02	29	0.78
	Barombi Mbo	43	431	122	632 (500/132)	10.36	0.56	3.12	0.09	8	0.60

^a^ before filtering in CoNet

All lake networks showed a quite comparable taxonomic profile, encompassing the same four major phyla (Proteobacteria, Firmicutes, Planctomycetes and Fusobacteria) (Fig. 1c), and six major families (Clostridiaceae, Pirellulaceae, Rhodobacteraceae, Fusobacteriaceae, Peptostreptococcaceae and Lachnospiraceae), with comparable node frequency (Additional files 2 and 3: Figures S1 and S2). Proteobacteria contributed with the largest number of nodes, typically followed by Firmicutes. All lake networks, except for L. Tanganyika, showed a large cluster of densely connected Firmicutes nodes (Fig. 1a, blue circles), which primarily belonged to the families Clostridiaceae and Peptostreptococcaceae (Additional file 2: Figure S1). In L. Tanganyika, this cluster was smaller and represented by eight nodes only (Fig. 1a). In general, major differences in lake network composition were seen for L. Tanganyika, which encompassed the largest cichlid phylogenetic and dietary diversity among all lakes (Additional file 1: Table S1). L. Tanganyika network was characterized by a substantially lower proportion of nodes belonging to the phylum Bacteroidetes and Firmicutes (Fig. 1c), particularly of families Clostridiaceae (5% of the nodes) and Lachnospiraceae (no nodes) (Additional file 3: Figure S2), and a higher representation of Actinobacteria and Verrucomicrobia. The network taxonomic profile (proportion of node per phylum) only partly reflected the microbiota taxonomic profile (proportion of OTUs per phylum in the original input matrices, i.e. 774 OTUs, including only most abundant OTUs); the latter was characterized by a remarkably homogeneous pattern of phyla representation across lakes and continents (Additional file 4: Figure S3). This indicates that the network composition does not simply mirror the OTU diversity of the input dataset.

**Fig. 2:**
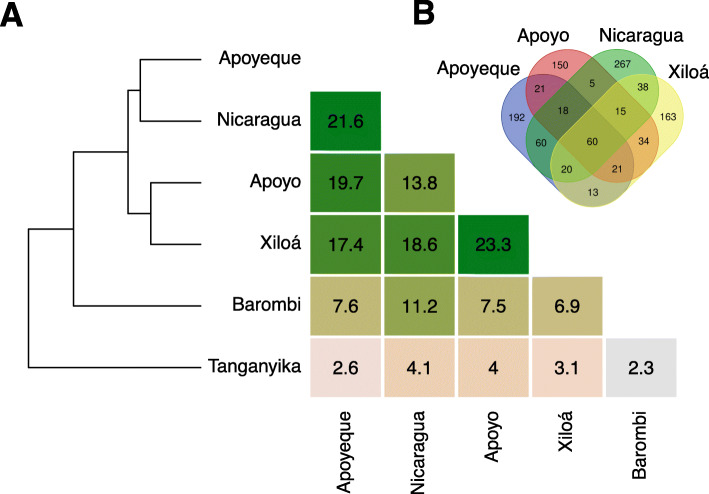
Distances among lake networks estimated by a Jaccard similarity index. **a** Heatmap of distance matrix values as proportion of shared OTU pairwise associations (i.e. edges) among lake networks and corresponding dendrogram. **b** The Venn diagram shows number of shared and unique associations among the four American lake graphs

We next explored similarities among individual lake networks based on shared pairwise associations as measured by the Jaccard index (where edges are taken as observations) (Fig. 2a). A total of 1797 unique associations were obtained across all lake networks. The four American lakes resembled each other more than any of the two African lakes, while these, Barombi Mbo and Tanganyika, did not cluster together. American lakes shared between 44 and 55% of their associations with any other lake network, with a total of 60 associations being shared across all four lakes (Fig. 2b). The closest resemblance was observed between crater L. Apoyo and Xiloá networks (sharing 23.3% of their total unique associations), followed by the similarity between lakes Nicaragua and Apoyeque (21.6%). On the other hand, the two African lakes did not cluster together and the large majority of associations were unique to each lake (i.e. 76% for crater lake Barombi Mbo and 87% for L. Tanganyika). Interestingly, Barombi Mbo network was more similar to any of the American lakes (6.9 to 11.2% of shared associations) than to L. Tanganyika (only 2.3% of shared associations), confirming findings based on node taxonomy (Fig. 1c). The distinctive profiles of the two African lakes are likely driven by the ecological and phylogenetic heterogeneity of these two datasets, both encompassing several cichlid genera and species and a wide range of dietary niches (see later for diet analyses).

### Core microbial associations

**Fig. 3:**
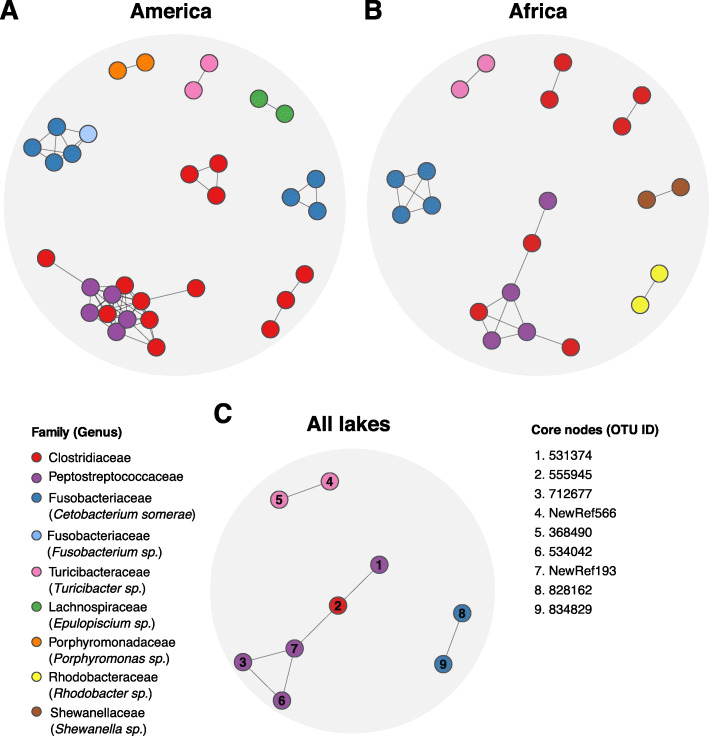
Core networks of common pairwise associations across geographical graphs. **a-c** Shared associations across the four American lakes (Nicaragua, Apoyo, Apoyeque and Xiloá; 60 edges, 32 nodes) (**a**), the two African lakes (Tanganyika and Barombi Mbo; 20 edges, 21 nodes) (**b**), and all lakes (7 edges, 9 nodes) (**c**). Nodes are colored according to the maximum taxonomic resolution achieved. Nodes in **c** are labelled by their OTU ID (right panel, for full taxonomic classification and abundance across samples, see Additional file 1: Table S2)

To explore whether specific pairwise associations were conserved across the range of cichlid geographical distribution, individual lake networks were intersected to retrieve the shared component (Fig. 3). To this goal, we generated a core network for America, by intersecting the four lake-specific networks (Fig. 3a), and a core network for Africa, by intersecting lakes Tanganyika and Barombi Mbo networks (Fig. 3b). We finally retrieved associations common to all networks (Fig. 3c).

The four American lakes shared 60 pairwise associations (32 nodes/OTUs) (Fig. 3a), structured into eight units (individual groups of nodes connected by at least one edge) of three phyla (Firmicutes, Fusobacteria and Proteobacteria) and six families. The major unit (12 nodes) included the two Firmicutes families, Clostridiaceae and Peptostreptococcaceae. Typically, all other units included associations among members of the same family (Clostridiaceae or Fusobacteriaceae), or the same genus (*Cetobacterium, Turicibacter, Epulopiscium* and *Porphyromonas*), except for one unit (with a *Cetobacterium*-*Fusobacterium* association*)* (Fig. 3a).

The two African lakes included 20 associations (21 nodes), structured into seven units (Fig. 3b), encompassing the same three phyla characterizing the American core and six families (Fig. 3a). Also in this case, the major unit (seven nodes) included the two Firmicutes families, Clostridiaceae and Peptostreptococcaceae, while the other units involved associations among members of the same family (Clostridiaceae) or genus (*Cetobacterium, Turicibacter, Shewanella,* and *Rhodobacter*).

The global core network, obtained through the intersection of all lake-specific networks, encompassed seven pairwise associations (nine OTUs) (Fig. 3c) that involved Clostridiaceae-Peptostreptococcaceae associations, and genus-specific associations between *Turicibacter* and *C. somerae* members*.* Three of the nodes (OTU-555945, OTU-712677 and OTU-828162) represented core OTUs (found in 90% of the specimens according to [24]; see Additional file 1: Table S2 for OTU counts and sample distribution). These associations can be considered as continent and lake- independent.

We note that the network intersections generated do not take into account indirect associations among OTUs, i.e. common nodes that are indirectly connected by means of few intermediate nodes (no direct edge connects the two nodes). Consequently, the size of the core could be potentially larger.

### Diet-specific network features

We next focused our analyses on the exploration of diet as a potential factor in driving dynamics in gut microbial association patterns. To exclude the geographic effect, we used the L. Tanganyika dataset which encompasses the largest diversity of cichlid trophic diversity (our dataset including carnivores, scale-eaters, omnivores, planktivores and herbivores) and provides a reasonable number of samples per dietary category for reliable network inference (unlike L. Barombi Mbo). We tackled this goal through a double approach: first, we built separate networks for the two most representative diets, herbivores (H) and carnivores (C; includes scale eaters) and compared properties. In the second approach, we mapped diet-associated features (i.e. OTU centered log-ratio (clr) transformed read abundances and effect sizes in pairwise diet contrasts) on the whole L. Tanganyika network.

**Fig. 4:**
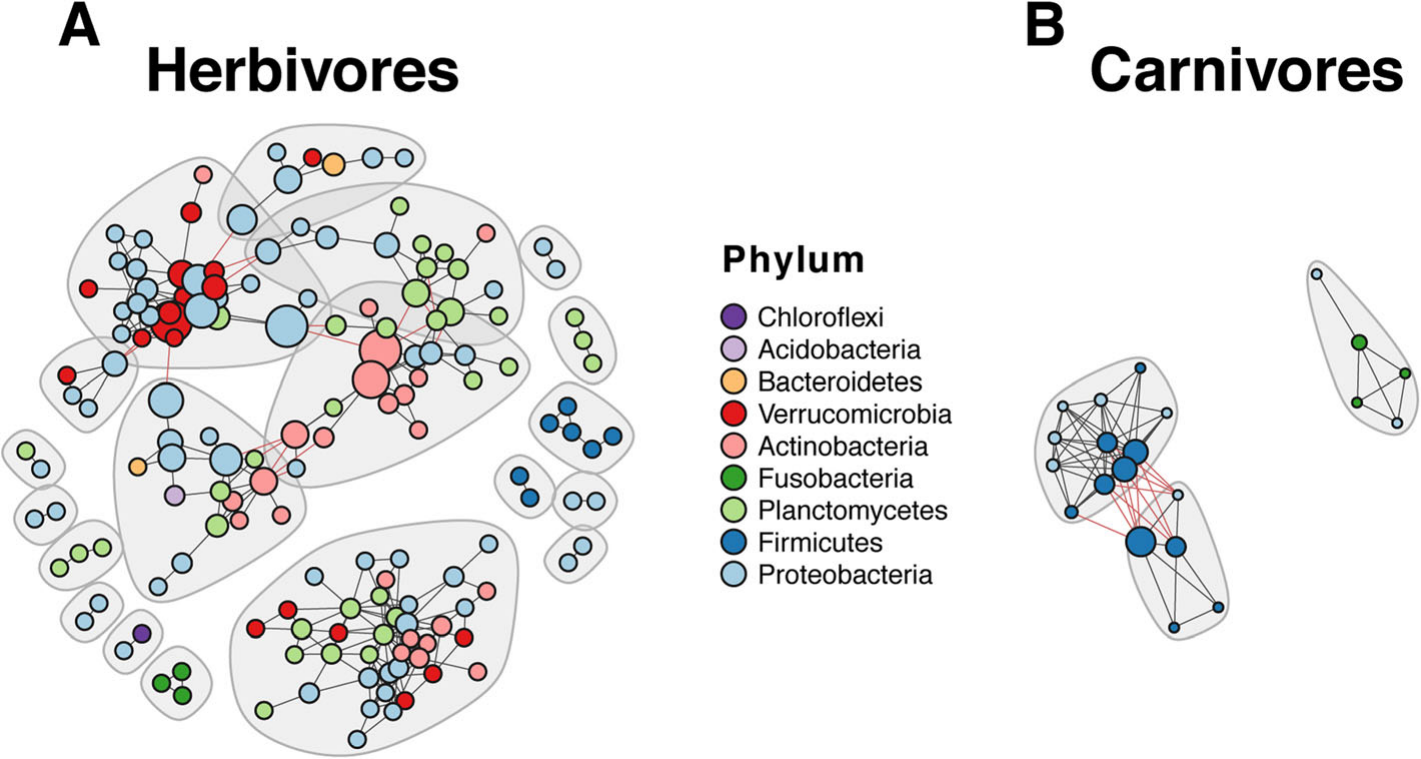
Diet-specific networks of L. Tanganyika herbivores and carnivores. Nodes are colored according to phylum and sized by betweenness values normalized by lake. Grey shades represent distinct modules (connected by red edges). Notice the higher complexity of the herbivore network (156 nodes and 339 edges; 19 modules) compared to the carnivore network (21 nodes and 70 edges; 3 modules)

The Tanganyika C and H networks showed remarkable differences in topology and node taxonomic representation (Fig. 4 for phylum and see Additional file 5: Figure S4 for family level), despite the use of a comparable sample size (*n* = 24 for C and *n* = 25 for H), similar OTUs richness in the input dataset (total number of observed OTUs = 460 for C and 542 for H) and same settings for network building (see Methods). The H network (156 nodes and 339 edges) was about eight-fold larger than the C network (21 nodes and 70 edges) and taxonomically more diverse, including nine phyla, the most conspicuous being the Proteobacteria (43%), followed by Planctomycetes (22%), Actinobacteria (15%) and Verrucomicrobia (11%) (Fig. 4a). Most represented families were Verrucomicrobiaceae, Rhodobacteraceae and Pirellulaceae (Additional file 4: Figure S3). Major hub nodes (according to normalized *betweenness*) involved members of the Proteobacteria (mostly of unknown families), Actinobacteria and Verrucomicrobia. In the C network (Fig. 4b), nodes encompassed a reduced diversity, involving the same three phyla constituting the African core network (see Fig. 3b). Most relevant hub nodes belonged to the Firmicutes, families Clostridiaceae and Peptostreptococcaceae (Additional file 5: Figure S4).

Modularity (according to Louvain) was ~ 2.5-fold higher in the H (0.72) than in the C network (0.29), with the H network accounting for 19 large modules (Fig. 4, grey-shaded areas) of heterogeneous taxonomic content, and the C network including three small modules only. The two networks shared only three associations. Still, edge randomization through node label shuffling, but preserving net topology, indicated that the number of shared edges was significantly higher than expected by chance (*p* = 0.005, 999 permutations). With respects to African core associations (Fig. 3b), C and H networks shared only five and four core associations, respectively.

**Fig. 5:**
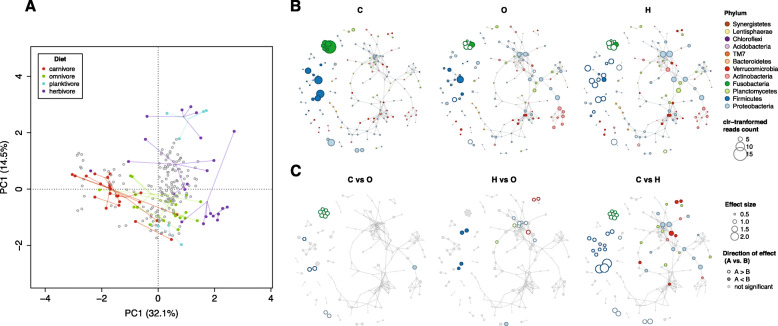
Mapping of diet contribution onto L. Tanganyika network. **a** Principal components analysis (PCA) of centered log-ratio (clr) transformed OTU counts for the Lake Tanganyika network. The PCA biplot shows both samples (i.e. individual fish, colored by diet), and OTUs (grey circles). Sample OTUs are grouped by cichlid species, as indicated by spider plots (lines connecting group samples to group centroid). The OTU coordinates in the PCA were used as a template to produce the network layout shown on panels **b** and **c**, as explained in the Methods. **b** Tanganyika network showing median node abundances for carnivorous (C), omnivorous (O) and herbivorous (H) diet. Node size is proportional to the median OTU abundance (after clr transformation) across individual specimens within each diet. Filled circles correspond to nodes with abundance above the geometric mean, whereas hollow circles indicate nodes with abundance below the geometric mean. **c** Tanganyika network showing node effect sizes for t-test pairwise comparisons between diets (estimated with ALDEx2). Nodes that significantly discriminated between diets (*p* < 0.05) are colored to phylum level, as in **b**. Hollow circles indicate nodes that tend to be relatively more abundant in the left-hand diet of the contrasted pair (e.g. C in C vs H), whereas filled circles show nodes that tend to be more abundant in the right-hand diet (e.g. H in C vs. H). Grey nodes were not significant

A diet effect on co-occurrence patterns was even clearer when we used the second approach, that is mapping diet-associated nodes on the L. Tanganyika network (Fig. 5). The PCA biplot representing samples (fish specimens) and features (OTUs, after clr transformation of OTU abundances) showed that the network microbial composition significantly segregated by diet (from carnivores through omnivores to herbivores, PERMANOVA, *p* < 0.001, excluding planktivores) (Fig. 5a). Within each diet, individuals tended to aggregate by species, indicating species-specific differences in gut microbiota composition. The two species classified as planktivores (a zoopanktonivore and a phytoplanktivore) were amply segregated in the PCA biplot. Being a mixed group, these were excluded from further analyses. We then mapped diet-associated nodes on the Tanganyika network by calculating the median clr-transformed OTU/node abundance across individual specimens within each diet (Fig. 5b). For this we used a network layout optimized to resemble the node layout emerging from the PCA analysis (see Methods; for the original PCA-based layout see Additional file 6: Figure S5). Results indicated a clear segregation across diets in terms of patterns of node (i.e. OTU) relative abundances (i.e. relative to the sample geometric mean) (Fig. 5b), with a significant increase in the number and taxonomic diversity of nodes from C to H, with omnivores (O) in between. Diet-associated nodes in C tended to form few modules of small size and homogeneous taxonomic content (i.e. most associations occurring between members of the same phylum). Both O and H showed a relevant increase in number of diet-associated nodes. More importantly, most of these diet-associated nodes were not scattered throughout the network, rather they grouped into a few large modules of heterogeneous taxonomic composition, mostly involving members of the phyla Proteobacteria, Verrucomicrobia, Planctomycetes and Actinobacteria (Fig. 5b). This transition was also fundamentally supported by fish specimen mapping on the network, with few outliers (Additional file 7: Figure S6).

The above pattern was further emphasized by large (> 1) and significant node effect sizes in pairwise comparisons among diets (Fig. 5c), supporting a strong contrasting pattern between C and H i.e. most of the nodes that were highly abundant in C were depleted in H (hollow circles) (*p* < 0.05). Compared to the C and O, the H were primarily characterized by a significantly higher representation of Verrucomicrobia and Proteobacteria nodes (Fig. 5c, red and blue circles).

## Discussion

We used the consortium of African and Central American cichlid fishes, with their remarkable diversification, to explore gut microbial associations through co-occurrence networks. Our main goals were 1) to understand how much of the observed gut microbial diversity translates into robust microbe-microbe associations, 2) whether there are conserved microbial co-occurrence patterns in cichlids, despite the scale of the host diversity, and 3) how microbial community structures adjust following changes in the host diet.

### Cross-lake network comparative analysis reveals community conservation

Major findings indicated that at least 21% (up to 43%) of total OTUs detected in individual cichlid lake assemblages (excluding the rare biosphere, see filtering in Methods) formed robust pairwise associations. Despite some lake-specific differences, notably in the L. Tanganyika network (but see later), these associations resolved into lake co-occurrence networks that were largely comparable in terms of both topology (Table 1), and taxonomic content (Fig. 1). American lakes formed particularly similar co-occurrence patterns, with a considerable overlap in terms of specific OTU associations (Fig. 2). Unlike the highly diverse African cichlid assemblages [17–19], the ecological and phylogenetic similarity of the American cichlids (they are all omnivores and belonged to the same genus *Amphilopus*) and their recent lake diversification [21] are likely to represent crucial factors in driving microbial network similarity, suggesting an interesting phylogeographic effect in shaping bacterial associations.

Persistent co-occurrences have been recently observed for free-living microbial communities, where a proportion of OTUs engage in similar interactions and form comparable communities in natural soils along large environmental gradients [13]. In host-associated microbiota, only a few sparse studies are beginning to explore the resilience and recurrence of similar association patterns across host populations [12], species [14], free versus laboratory-reared cultures [14], and experimental treatments [15]. In humans in particular, specific associations and network features (including modules) were found to be conserved across populations, suggesting that despite considerable intraspecific microbial variation and geographical distance, the microbiome tended to organize into stable interactions [12].

In our study, we observed network taxonomic and topological properties conservation at a macroscale level, i.e. across gut communities encompassing different host species within a single family (the Cichlidae), and along a broad phylogeographic and ecological gradient. Such conservation of microbial association patterns indirectly supports the presence of common constraints in the process of gut community assembling in cichlids, putatively driven by conserved aspects of the cichlid gut environment, including physiology and immune system, that can favor the retention of specific microbes and their persistence in the gut. In line with these findings, recent studies in fishes have shown a predominant role of host selection in shaping gut community composition, with deterministic rather than stochastic processes appearing to guide the early onset of the gut microbiota assembly [2, 27]. This scenario could also explain the similarities in association patterns observed across the distinct cichlid assemblages, although experimental studies are clearly needed to validate their biological relevance and understand the community ecological processes taking place in the cichlid gut.

Intriguingly, a small set of associations (seven) was robust to all data partitioning, being consistently found in all the lake networks (i.e. core, Fig. 3c). Particularly, members of the families Clostridiaceae and Peptostreptococcaceae (phylum Firmicutes), involved in five core associations, were also forming consistent co-occurring pairs in all lake and continental networks, where they typically resolved into large modules (Fig. 1a-b). Whether these modules represent ecologically or functionally equivalent units across largely distinct systems clearly needs further investigation. Similarly, recurring pairwise associations were also observed between members of the genus *Turicibacter*, a common inhabitant of animal guts, and between members of the species *C. somerae* (Fig. 3c). The latter formed few conserved modules in each lake network, suggesting putative niche overlapping and/or in situ diversification. *C. somerae* is a supplier of vitamin B12 (also known as cobalamin) [28] and represents the most conspicuous member of the cichlid gut, where it showed a systematic presence in all specimens [4, 24]. Recent studies have shown that cobalamins are essential nutrients for both animals and microorganism growth and metabolism, dictating important metabolic dependencies among them [29].

The nature and functional relevance of all these persistent associations remain unclear. An important aspect to consider is the large phylogeographical scale of network comparison in this study, which may be more relevant to detect association patterns at higher levels of bacteria hierarchical organization (from genera to families/orders), driven by ecological similarity within taxonomic group [30], rather than among OTUs. A puzzling question is how the same co-occurring pairs of OTUs are established in these highly diverse lake assemblages. We note that vertical or restricted transmission of bacteria over time can also generate patterns of co-occurrence, and this is an avenue that is worth considering. On this line, both Peptostreptococcaceae and *Turicibacter*, here members of the cichlid core network, were shown to be highly heritable in mice and humans [31]. Undoubtedly, the biogeography of these widespread OTUs and their ecological role need to be fully investigated if we are to understand their co-occurrence patterns in cichlids.

### Microbial community shift along dietary changes

While common traits in cichlids might be responsible for general conservation of gut microbial community aspects, we expected diet to represent a major factor in driving community changes. Despite the extensive literature supporting a role of diet in changing the compositional aspects of the gut microbiota, little attention has been paid to changes in community association patterns (for an example outside humans, see [15]). We had previously shown that diet was a key factor shaping the taxonomic content of the African cichlid gut microbiota, with the herbivore-type (H) gut microbiota being characterized by a significantly higher taxonomic diversity compared to the carnivore-type (C) one [4]. Here we showed that the microbial compositional diversity associated to these two extreme dietary categories in L. Tanganyika cichlids resolved into highly distinct co-occurrence networks (Fig. 4). In particular, the complex H network was larger in size, modularity and taxonomic diversity, compared to the smaller C network. A similarly important change in community structure was observed by mapping diet-specific node size on the whole Tanganyika network, revealing a clear contrasting pattern of node abundances between C and H (Fig. 5b-c). These differences can be partly associated to the distinct metabolic requirements of the two diets and the potential role of the gut microbiota in facilitating dietary adjustments [32]; in herbivores in particular, bacteria are essential players in the digestion of fibers and can establish complex metabolic interdependencies [33]. Interestingly, the same phyla that we previously showed to be significantly overrepresented in the African herbivores [4] (Verrucomicrobia, Proteobacteria, Actinobacteria and Planctomycetes) were found here to form major clusters of positive associations (Figs. 4 and 5). While network analyses alone cannot presently discriminate between microbial coexistence driven by metabolic interdependencies or shared niche preference, these clusters of co-occurring taxa/OTUs provide interesting candidates for future studies seeking to understand the role of the gut microbiota in the evolution of fish herbivory. At present, the functional profiles of these bacteria remain unresolved, as most of the OTUs involved are novel and show poor taxonomic resolution beyond the family level. Finally, fish accessibility to bacterial taxa involved in co-occurrence patterns needs to be explored. Recent studies suggest that diet-specific taxa could be simply sourced from common dietary inputs. The macroalgal microbiota, for instance, is known to be significantly enriched in algal polysaccharide-degrading bacteria in comparison to the water column [34]. This could hypothetically explain patterns of microbial co-occurrence across the diverse algal consumers from L. Tanganyika, a scenario that requires further investigation.

## Conclusions

Altogether our study illustrates the power of co-occurrence networks to begin exploring community aspects of animal-associated microbiota, particularly in wild animals. Cross-lake network analyses of the gut microbiota identified important network commonalities in otherwise highly diverse fish assemblages. This supports the existence of major constraints in microbial community assembly and maintenance in cichlids. By excluding the geographical effect, we also identified specific microbial associations and hub taxa potentially involved in key dietary transitions within a lake.

Overall, our findings provide that very first exploration of the factors shaping microbial association patterns in the cichlid gut. Inclusion of additional microbial community data, both from the host and the environment, is required to support the above network inferences and validate specific association patterns. Furthermore, the fish phylogenetic diversity should also be integrated into the network structure; this could be tackled, for example, by comparison of multiple species-specific co-occurrence networks, by means of an extensive fish sampling within a single species. Finally, ongoing lab cultures are now targeting key community changes between cichlid specimens raised under high and low-fiber content diets (data in preparation). This could prove useful to test the predictive role of the hub taxa and association patterns here identified in driving gut community changes, as well as to explore bacteria functional relevance.

## Materials and methods

### Sampled data and network generation

Samples corresponded to our previously published dataset [24] and included a total of 277 specimens (42 species) sampled across nine lakes from Africa and America: the two African lakes, Tanganyika (*n* = 73) and Barombi Mbo (Cameroon, *n* = 43) and the seven Central American lakes, Apoyo (*n* = 25), Apoyeque (*n* = 25), Xiloá (*n* = 43), Masaya (*n* = 14), Asososca León (*n* = 13), Nicaragua (*n* = 29), and Managua (*n* = 12), all found in Nicaragua. Except for the large lakes Tanganyika, Nicaragua and Managua, all others are small crater lakes. African specimens encompassed multiple genera and dietary niches, including carnivores, scale-eaters, omnivores, planktivores and herbivores, while American specimens belong to a single genus (*Amphilopus*) and are largely omnivores (see [24] for details on diet assignment). Gut microbiota data was obtained through three 16S rRNA Miseq runs using the same protocol for DNA extractions, library preparation and sequencing. Briefly, DNA was extracted from whole intestines using the protocol described by [35]. The region V3-V4 of 16S rRNA was amplified for each sample fish in three-replicates with primers S-D-Bact-0341-b-S-17 and S-D-Bact-0785-a-A-21 [36]. Amplicons were barcoded, pooled and equimolar libraries were sequenced on the Illumina MiSeq v.3 instrument (600 cycle cartridge, 300 bp paired end, San Diego, CA, USA) with 10% PhiX, at the Center for Genomic Regulation in Barcelona (Spain). The use of the same experimental approach and sequencing in three Miseq runs minimized technical artifacts in driving community structure similarity. After quality filtering and reads merging, sequences from the three runs (18287373) were combined and filtered according to OTU taxonomy, abundance and frequency across samples (see [24] for details). We finally obtained an OTU abundance table including 3639 OTUs and 16,083,429 total counts. This table was further filtered to remove OTUs contributing < 0.005% of total counts across all samples, reducing the dataset to 774 OTUs and 15,467,721 total counts (provided as Additional file 1: Table S2). To account for the heterogeneity of the data, the OTU matrix was then split into several datasets according to geographical (continent and lake) and ecological variables (diet), setting an arbitrary minimum number of samples for network generation (*n* = 20 fish specimens). For the American lakes Masaya, Managua and Asososca León, where *n* < 20, sample contribution was integrated into the American continental network.

Each dataset (abundance matrix) was loaded into the CoNet ensemble app available in Cytoscape v. 3.7.2 [25] for network generation (where rows are OTUs and columns are fish specimens). Settings were the same for lake and continental datasets. Specifically, the data was filtered for OTU occurrence across samples according to the minimum value suggested by the program, keeping the sum of filtered rows (row minimum occurrence above 31% for all lake/continental datasets). This step minimizes sparsity issues and false correlations due to double zero problem when computing similarity or distance coefficients using species presence-absence or abundance data [25, 37]. In all cases, data was normalized by column (col_norm; entries divided by column sum) to reduce compositionality issues associated to different sampling efforts. Four correlation and dissimilarity metrics were chosen: Spearman, Pearson, Bray-Curtis dissimilarity and Kullback-Leibler dissimilarity, with correlation thresholds set to retain 1000 edges (top positives and negatives) that were supported by all four metrics (score = mean). Edge significance was tested through 1000 permutations and bootstraps (method =” brown”), retaining edges with merged *p*-values < 0.05 after Benjamini-Hochenberg’s correction (q-value).

The impact of diet on network topology and properties was explored on the Lake Tanganyika dataset as it encompassed the largest diversity of diets among all lakes and is the only lake that includes herbivore cichlids. The Tanganyika dataset was further split into herbivores (*n* = 25) and carnivores plus scale-eaters (here grouped into the same category) (*n* = 24) (see Additional file 1: Table S1). CoNet settings for dietary analyses were chosen to be more conservative: minimum OTU occurrence was set to 60% of the samples for both herbivores and carnivores, considering copresence only to reduce potential spurious correlations due to matrix sparsity. For both carnivores and herbivores, we retained the top 1000 positive edges resulting from the intersection of the four metrics. For the carnivore network this resulted in the inclusion of all edges, providing that total number of edges for this network was less than 1000.

### Network analysis

Each network was loaded into R for all subsequent analyses. Network properties, including simple and complex parameters of global net and node topologies were estimated with R package *igraph* version 1.2.5 [26]. Number of modules, defined as clusters of nodes that form coherent structural subsystems of interacting units, and modularity (M) were measured according to the Louvain algorithm, with the function *cluster_louvain* in the *igraph* package [38]. Hub nodes (OTUs) were explored by measures of normalized betweenness centrality with function *betweenness* in the *igraph* package. Distances among lakes networks were estimated by a Jaccard similarity index, taking edges as observations and building a distance matrix based on edge presence/absence among lakes. Distances were graphically represented through a dendogram using the function *hclust* in base R.

The cichlid core was defined as the fraction of edges that were conserved across all lake networks. Shared edges were obtained through the function *intersection* in the *igraph* package.

To explore how fish diet mapped onto the Lake Tanganyika network, we first examined whether network OTU content differed among diets. Since OTU reads are compositional [39], we first processed the data using the centered log-ratio (clr) transformation, that is, for a given sample (fish specimen), OTU counts were first divided by the sample geometric mean and then this ratio was log transformed. Since zero counts are not allowed because of logarithms, data were preprocessed to impute zeros with the count zero multiplicative method using function *cmultRepl* in the *zCompositions* package [40]. The resulting clr-transformed counts are suitable for principal components analysis (PCA), with the implicit distance being the Aitchison distance, i.e., the Euclidean distance among clr-transformed data [41]. PCA allows for a natural joint representation of samples (fish specimens) and features (OTUs) in reduced dimension, the biplot. Separation among diets was tested using PERMANOVA, with diet as the factor of interest and fish species as a nested fixed factor (because samples are aggregated by fish species), followed by pairwise contrasts among diets. All PERMANOVA analyses were done with function *adonis* in the *vegan* R package [42] using the Euclidean distance on clr-transformed data (i.e., the Aitchison distance).

Once it was established that OTU assemblages differed among diets, we looked at which OTUs best discriminated among diets in pairwise comparisons. For this, we used ANOVA-like differential expression (ALDEx) analysis with the ALDEx2 R package [43]. Specifically, we used function *aldex.effect* to calculate OTU effect sizes (median of the ratio of the between diet difference and the larger of the variance within the two diets being compared), and function *aldex.ttest* to obtain Benjamini-Hochberg corrected *p*-values for each OTU from Welch’s t-tests. For each diet pairwise comparison, we showed both effect sizes and significance (*p* < 0.05) mapped onto the Tanganyika network as explained next.

Whereas the PCA biplot offers a natural layout for displaying the Tanganyika network and mapping diet features onto it, it is not optimal for visualizing important network features such as edges, components and hubs. On the other hand, common network layout algorithms, such as the Fruchterman and Reingold force-directed algorithm (FR, [44]) are optimal for displaying network features, but are blind to spatial relations among added mapped features (diet, in our case). In order to add the latter, we mapped diet features onto an FR layout that approached as much as possible the OTU layout produced by the PCA. To do so, we produced 5000 random FR layouts and chose the seed for the pseudorandom number generator that produced the layout with the smallest sum of squared distances between the FR layout and the PCA layout. For this, we used function *procrustes* in the *vegan* package. This has the added benefit that the resulting layout is also rotated and orientated similarly to the target layout (i.e., the PCA layout). The chosen layout was used to display both median clr-transformed OTU counts by diet and effect sizes in pairwise comparisons. For reference, the clr-transformed counts mapped onto the original PCA layout are provided in Additional file 6, Figure S5.

## Supplementary information

**Additional file 1: Table S1.** Sample metadata; **Table S2.** OTU input matrix (774 OTUs) and taxonomic classification.

**Additional file 2: Figure S1.** Individual lake networks with nodes colored by family level. For clarity, only the first 36 most abundant families are color-coded (remaining families are labelled as “Others” and shown in white). The same color is repeated across different families but in association to distinct border colors (i.e. black, gray and white).

**Additional file 3: Figure S2.** Taxonomic composition of continental and lake networks as proportion of nodes per family.

**Additional file 4: Figure S3.** Microbiota taxonomic composition by lake and continental datasets expressed as proportion of OTUs present in the original input matrices (774 OTUs after filtering out low abundant OTUs).

**Additional file 5: Figure S4.** Diet-specific networks of L. Tanganyika herbivores and carnivores. Nodes are colored according to family and sized by betweenness values normalized by lake. Grey shades represent distinct modules (connected by red edges).

**Additional file 6: Figure S5.** Mapping of diet contribution onto the L. Tanganyika co-occurrence network based on the original PCA layout. Node size displays median clr-transformed OTU counts by diet, with filled (hollow) nodes signifying OTUs that are more (less) abundant than the sample geometric mean, as in Fig. 5.

**Additional file 7: Figure S6.** Mapping of individual cichlid species onto the Tanganyika network. The network layout is the same as in Fig. 5, and node circle size is proportional to median clr-transformed OTU abundances in individuals belonging to the same species. Species are ordered by diet (color coded) and alphabetically within a diet. Only species with two or more representative specimens are shown.

## Data Availability

The microbiota data used in this study are available at Bioproject PRJNA531389 (American dataset) and PRJNA341982 (African dataset).

## Abbreviations

OTU: Operational taxonomic unit
C: Carnivore
H: Herbivore
O: Omnivore
clr: Centered log-ratio
PCA: Principal component analysis
FR: Fruchterman and Reingold
