# Supplementary figures and images for: Microbial co-occurrence networks of gut microbiota reveal community conservation and diet-associated shifts in cichlid fishes
Source: Anim Microbiome. 2020 Sep 29;2:36. doi: 10.1186/s42523-020-00054-4 (PMC7807433; doi:10.1186/s42523-020-00054-4)

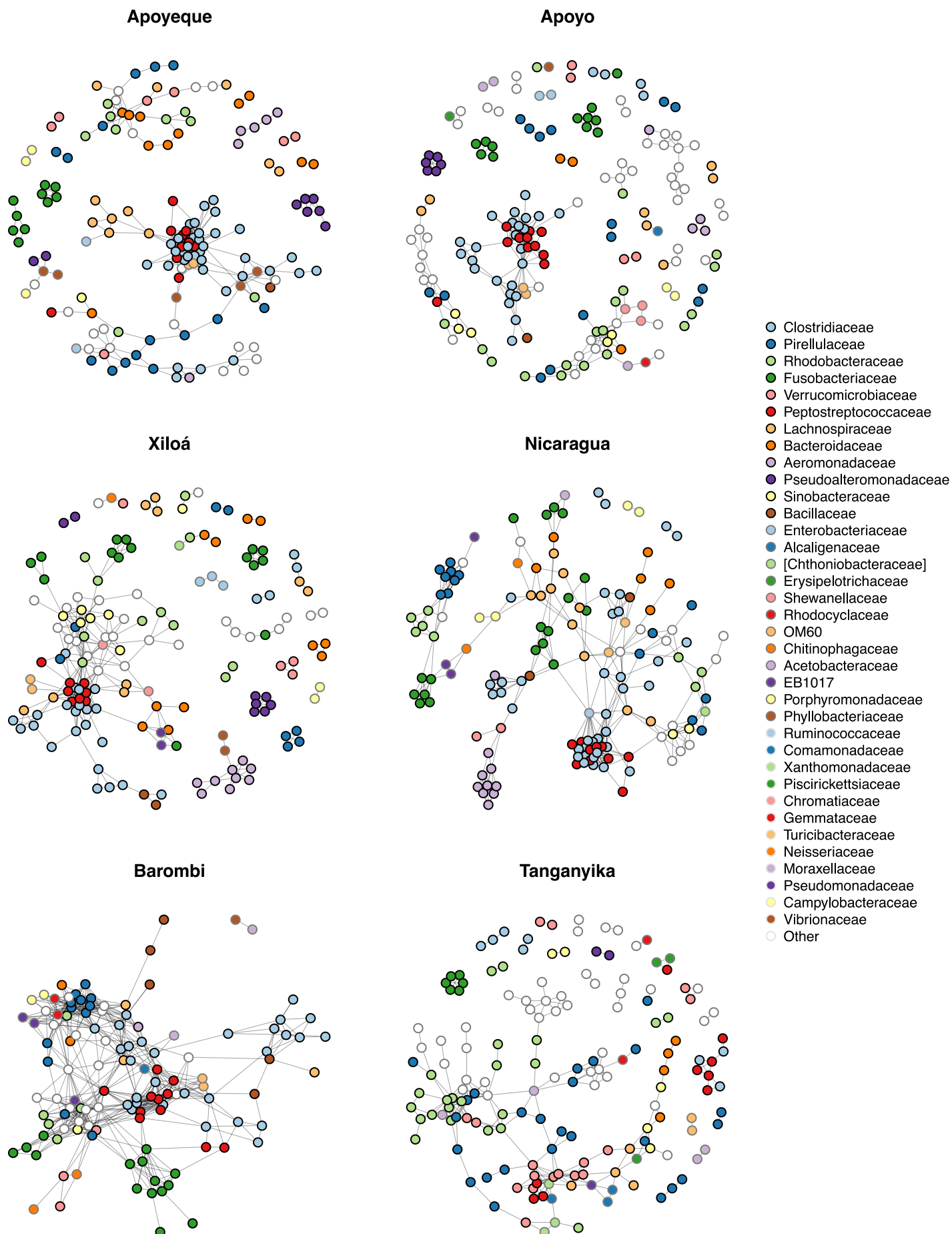

**Fig. S1**

Supplement: Supplementary file 2 — Additional file 2: Figure S1. Individual lake networks with nodes colored by family level. For clarity, only the first 36 most abundant families are color-coded (remaining families are labelled as “Others” and shown in white). The same color is repeated across different families but in association to distinct border colors (i.e. black, gray and white). [file 42523_2020_54_MOESM2_ESM.pdf]

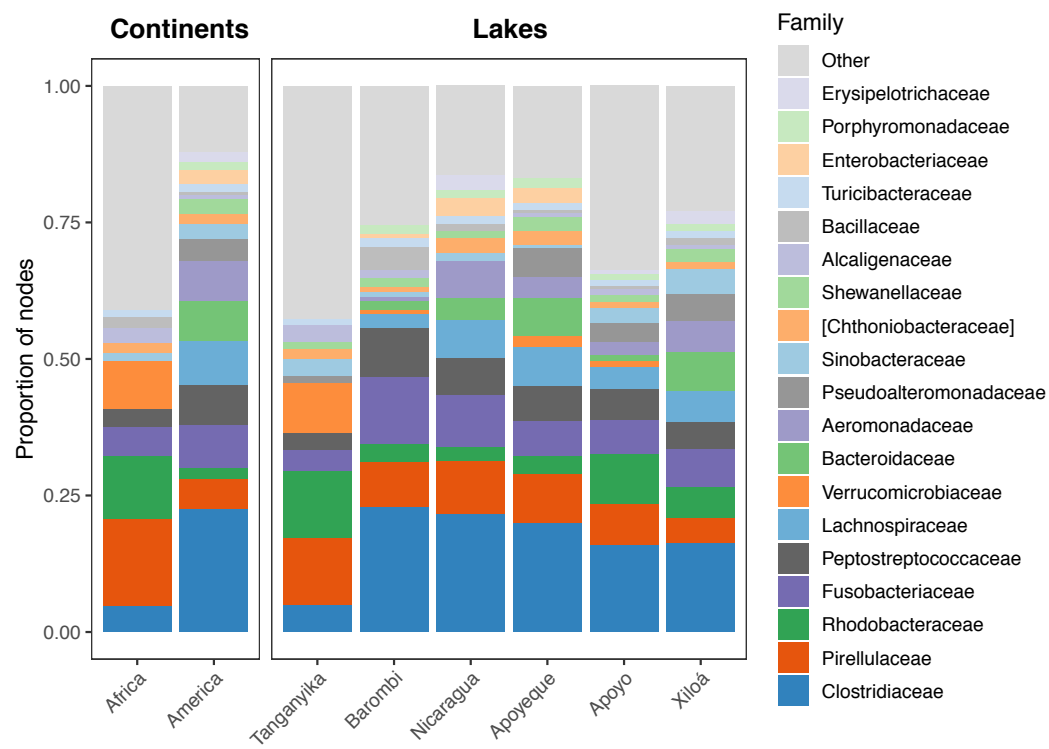

Fig. S2

Supplement: Supplementary file 3 — Additional file 3: Figure S2. Taxonomic composition of continental and lake networks as proportion of nodes per family. [file 42523_2020_54_MOESM3_ESM.pdf]

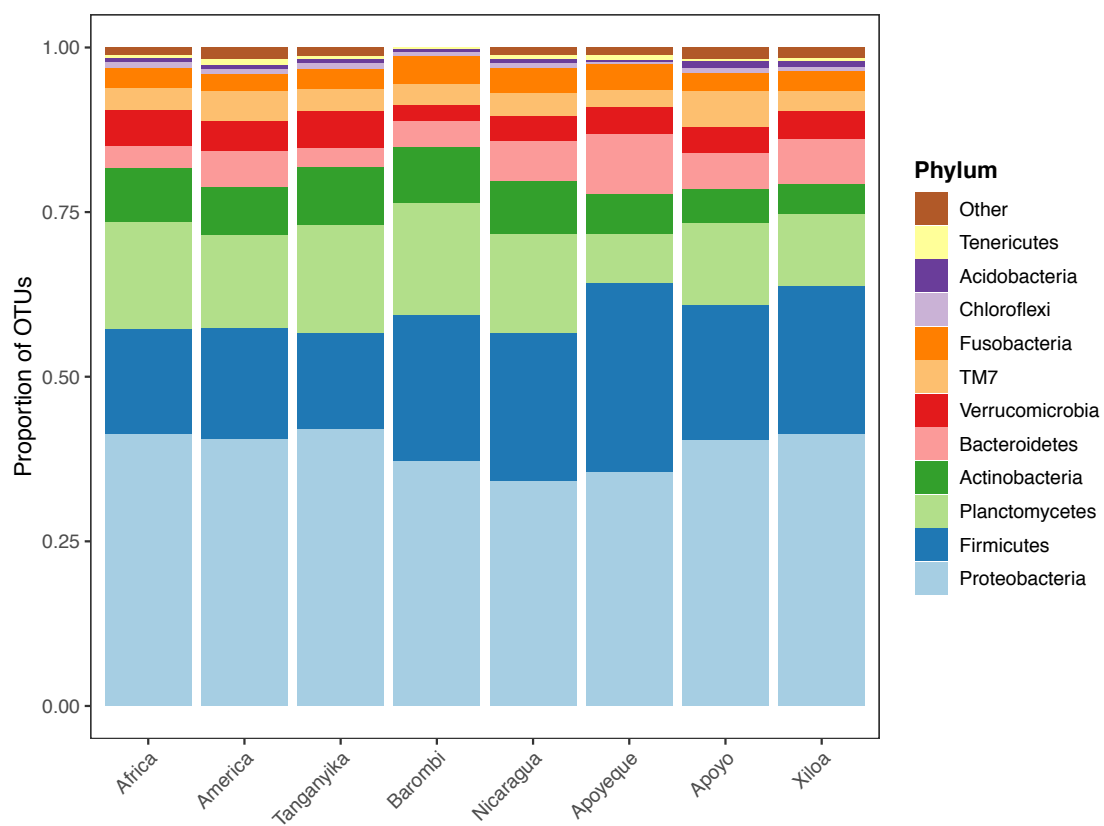

Fig. S3

Supplement: Supplementary file 4 — Additional file 4: Figure S3. Microbiota taxonomic composition by lake and continental datasets expressed as proportion of OTUs present in the original input matrices (774 OTUs after filtering out low abundant OTUs). [file 42523_2020_54_MOESM4_ESM.pdf]

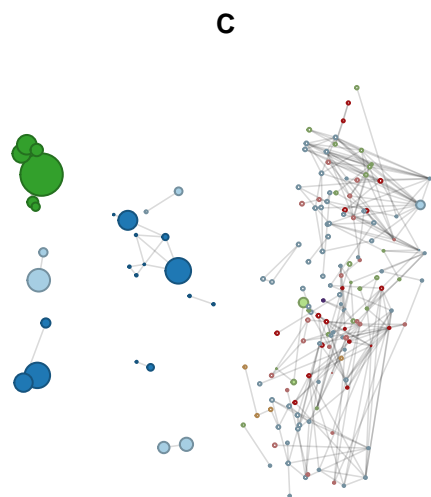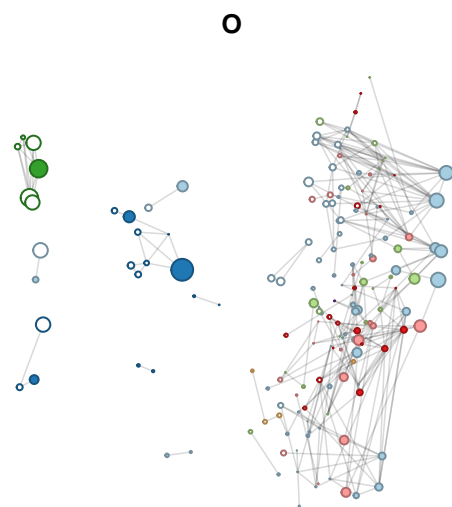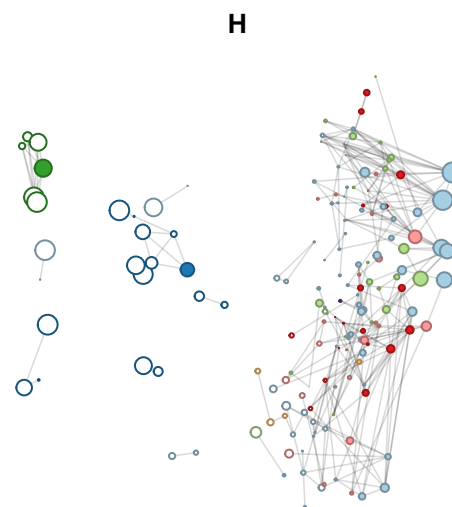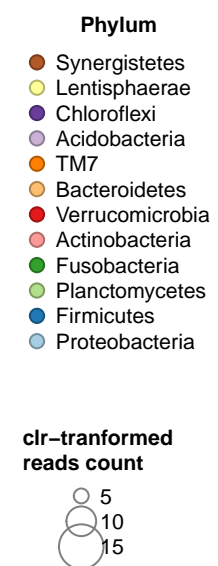

Supplement: Supplementary file 6 — Additional file 6: Figure S5. Mapping of diet contribution onto the L. Tanganyika co-occurrence network based on the original PCA layout. Node size displays median clr-transformed OTU counts by diet, with filled (hollow) nodes signifying OTUs that are more (less) abundant than the sample geometric mean, as in Fig. 5. [file 42523_2020_54_MOESM6_ESM.pdf]
