# Supplementary material for: Microbial co-occurrence networks of gut microbiota reveal community conservation and diet-associated shifts in cichlid fishes
Source: Anim Microbiome. 2020 Sep 29;2:36. doi: 10.1186/s42523-020-00054-4 (PMC7807433; doi:10.1186/s42523-020-00054-4)
Supplement: Supplementary file 5 — Additional file 5: Figure S4. Diet-specific networks of L. Tanganyika herbivores and carnivores. Nodes are colored according to family and sized by betweenness values normalized by lake. Grey shades represent distinct modules (connected by red edges). [file 42523_2020_54_MOESM5_ESM.pdf]

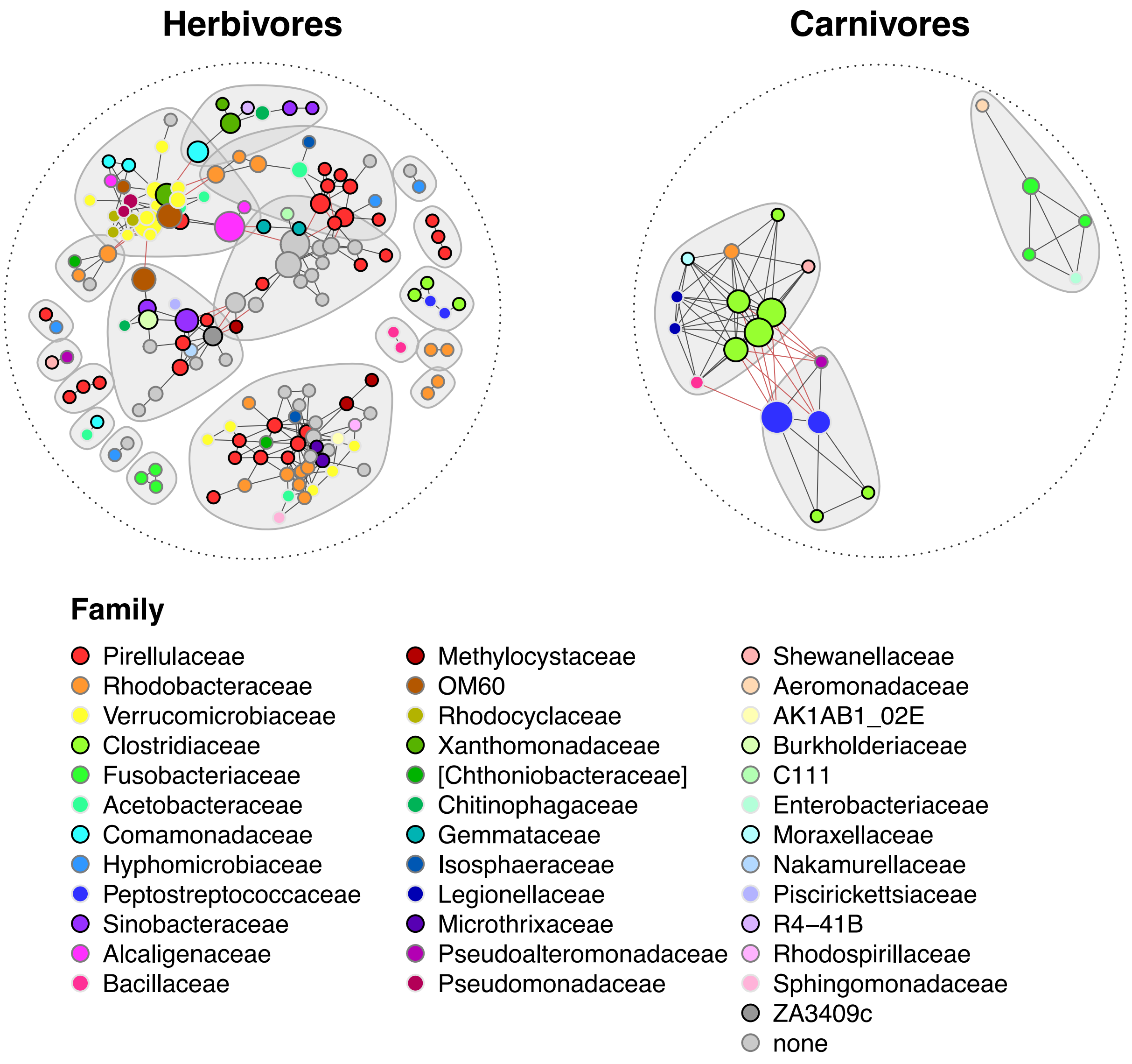
Herbivores
Carnivores
Family
Pirellulaceae
Rhodobacteraceae
Verrucomicrobiaceae
Clostridiaceae
Fusobacteriaceae
Acetobacteraceae
Comamonadaceae
Hyphomicrobiaceae
Peptostreptococcaceae
Sinobacteraceae
Alcaligenaceae
Bacillaceae
Methylocystaceae
OM60
Rhodocyclaceae
Xanthomonadaceae
[Chthoniobacteraceae]
Chitinophagaceae
Gemmataceae
Isosphaeraceae
Legionellaceae
Microthrixaceae
Pseudoalteromonadaceae
Pseudomonadaceae
Shewanellaceae
Aeromonadaceae
AK1AB1_02E
Burkholderiaceae
C111
Enterobacteriaceae
Moraxellaceae
Nakamurellaceae
Piscirickettsiaceae
R4−41B
Rhodospirillaceae
Sphingomonadaceae
ZA3409c
none

Fig. S4
