# Supplementary material for: Microbial co-occurrence networks of gut microbiota reveal community conservation and diet-associated shifts in cichlid fishes
Source: Anim Microbiome. 2020 Sep 29;2:36. doi: 10.1186/s42523-020-00054-4 (PMC7807433; doi:10.1186/s42523-020-00054-4)
Supplement: Supplementary file 7 — Additional file 7: Figure S6. Mapping of individual cichlid species onto the Tanganyika network. The network layout is the same as in Fig. 5, and node circle size is proportional to median clr-transformed OTU abundances in individuals belonging to the same species. Species are ordered by diet (color coded) and alphabetically within a diet. Only species with two or more representative specimens are shown. [file 42523_2020_54_MOESM7_ESM.pdf]

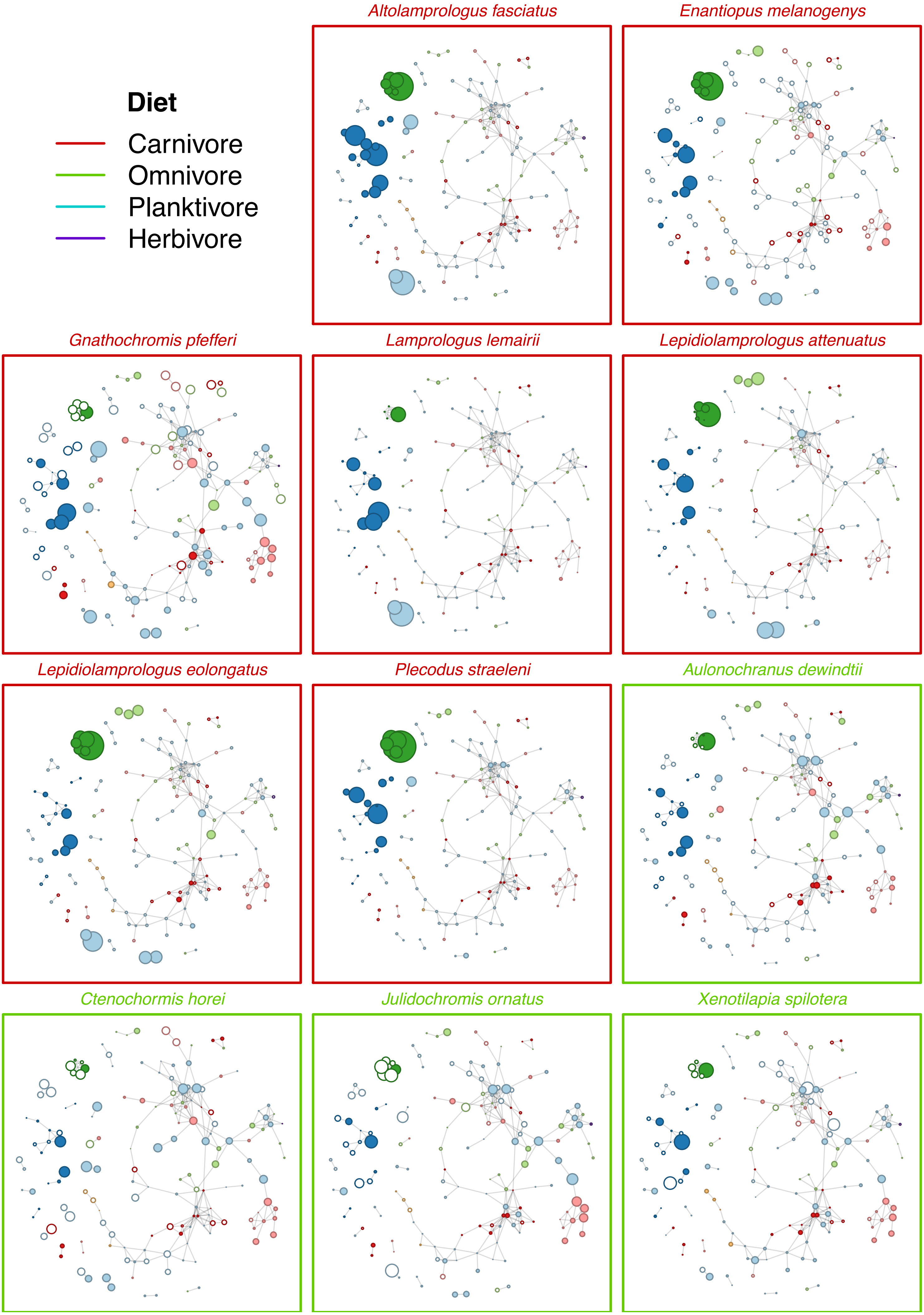
Diet
Carnivore
Omnivore
Planktivore
Herbivore
Altolamprologus fasciatus
Enantiopus melanogenys
Gnathochromis pfefferi
Lamprologus lemairii
Lepidiolamprologus attenuatus
Lepidiolamprologus eolongatus
Plecodus straeleni
Aulonochranus dewindtii
Ctenochormis horei
Julidochromis ornatus
Xenotilapia spilotera

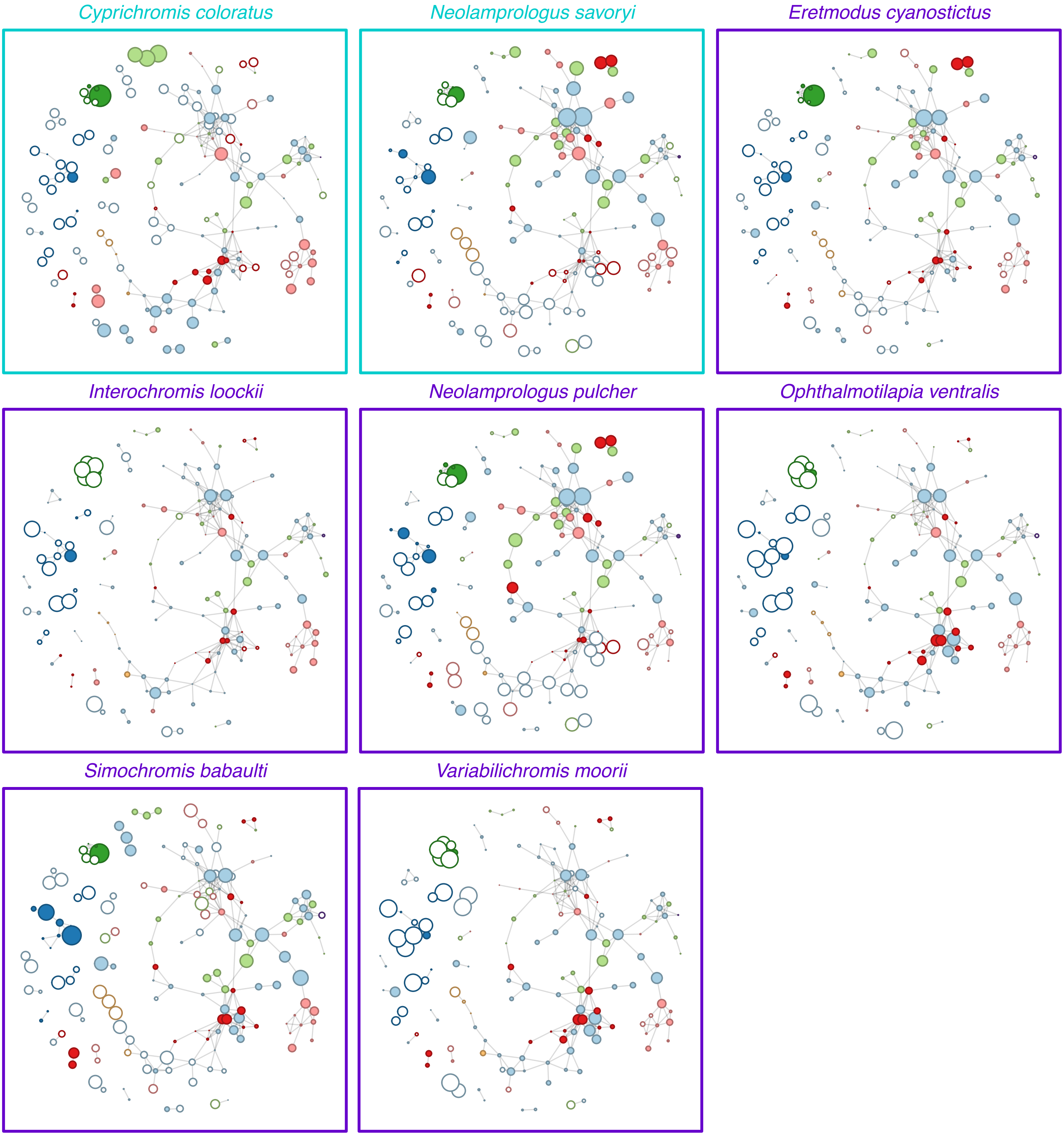
Cyprichromis coloratus
Neolamprologus savoryi
Eretmodus cyanostictus
Interochromis loockii
Neolamprologus pulcher
Ophthalmotilapia ventralis
Simochromis babaulti
Variabilichromis moorii
